# Pineapple by-products: A critical review of their bioactive compounds as eco-friendly pesticides in pest management

**DOI:** 10.1016/j.fochx.2025.102567

**Published:** 2025-05-19

**Authors:** Benjamin Bonsu Bruce, Isaac Duah Boateng

**Affiliations:** a Council for Scientific and Industrial Research (CSIR), Plant Genetic Resources Institute, P.O. Box 7. Bunso, Eastern Region, Ghana; b Organization of African Academic Doctors, P. O. Box 25305-00100, Nairobi, Kenya; c Certified Group, 199 W Rhapsody Dr, San Antonio, TX 78216, USA.

**Keywords:** Pineapple waste, Bioactive compounds, Polyphenols, Eco-friendly pesticides, Pests and pathogens

## Abstract

Pineapple processing generates significant waste in the form of peels, leaves, crowns, stems, and cores. Although their bioactive compounds have been explored, a literature review on their pesticidal properties is lacking. This paper reviews the research on the bioactive compounds in the byproducts (peels, leaves, stem, crown and core) in the last 5 years to give the recent knowledge on the bioactive compounds of the pineapple byproducts as ecofriendly pesticides in food storage management, examining their efficacy against common pests, mechanisms of action, and potential applications while considering human health safety and environmental impact. Among the bioactives identified in the pineapple waste are phenolic, terpenes, and organic acids. Studies have highlighted the pesticidal properties of extracts from waste against various food storage pests. They disrupt pest physiology, inhibit growth, and have antimicrobial activity. Despite these studies over the last five years, there are various opportunities to research the bioactive compounds present in pineapple waste, which could drive its commercialization and utilization.

## Highlights

• Pineapple by-product ‘s bioactive compounds’ pesticidal properties were reviewed.
• Bioactives identified in the byproduct are phenolic, terpenes and organic acids.
• Pesticidal activities of extracts from the byproduct is effective against pests.
• Studies into various bioactive compounds present in the byproduct to drive its utilization is needed.

## 1 Introduction

Pest and disease management in food storage is a crucial element that is sometimes overlooked within the complex network of food production and distribution. Pests not only endanger the safety of stored food but also result in substantial economic losses. Climate change is exacerbating pest infestations. Therefore, it is becoming more important to prioritize the safety of stored food. The use of chemical pesticides in food storage pest and disease management has been a common practice for decades. While these conventional pesticides are effective in controlling pests, they pose significant risks to human health and the environment (Kaur et al., 2021; Khan et al., 2023; Shekhar et al., 2024; Sinha et al., 2024). Prolonged exposure to chemical residues in food products can lead to serious health effects, including acute poisoning and chronic diseases (Kalyabina et al., 2021). Identifying perilous chemical pesticide residues in foods and growing consumer knowledge of food safety have resulted in restricting certain pesticides, and plant-based insecticides are becoming popular in organic agriculture (Ahmad et al., 2024).

In response to these challenges, there has been an interest in the development and adoption of eco-friendly alternatives to conventional pesticides in food storage pest management (Ben Mrid et al., 2021). Natural sources such as plants, microbes, minerals, and certain animal products serve as the source of eco-friendly pesticides, also referred to as biopesticides or organic pesticides. Unlike chemical pesticides, eco-friendly pesticides offer several advantages, including biodegradability, minimal toxicity to humans, and a healthy environment. Researchers have posited that natural products exhibit non-persistence due to their inherent biodegradability (Sharma et al., 2020). The significance of biopesticides in food storage pest and disease management extends beyond their environmental and health benefits. These sustainable pest management solutions play a crucial role in ensuring food security and preserving the quality and integrity of food products. Pest infestations in food products can result in significant economic losses due to damage to stored crops, contamination of food products, and regulatory non-compliance.

Botanicals are currently used in various industries, including fragrance and cosmetics, agriculture, pharmaceuticals, food, and beverages. Several studies have previously highlighted the importance and role of bioactive components (carvacrol, menthol, and limonene) as effective and helpful antibacterial, herbicidal, pesticidal, and antioxidant agents in the agri-food business (Boukhatem et al., 2020). There is a growing interest in exploring sustainable alternatives to conventional pesticides in the food and agriculture industries, such as those used in dairy, grains, and vegetables (e.g., maize, eggplants, cabbage). One such avenue of investigation involves utilizing bioactive compounds derived from pineapple waste as potential pesticides.

Pineapples (*Ananas comosus*) are perennial herbaceous plants that are cultivated on coastlines and in tropical regions, including China, Indonesia, South Africa, Malaysia, Nigeria, Costa Rica, the Philippines, and Thailand (Fouda-Mbanga & Tywabi-Ngeva, 2022). Pineapples are cultivated on over a million hectares of soil, generating $ 9 billion for the global economy each year (Chen et al., 2021; Yabor et al., 2020). Four major varieties of Pineapple are available worldwide: Red Spanish, Smooth Cayenne, Abacaxi, and Queen (Salve & Ray, 2020). Pineapple and its derivatives are popular due to their pleasant aroma and flavour. The economic importance of the pineapple species has been recognized in relation to its gross production value (Jatav & Bhattacharya, 2022). The processing of pineapple fruit generates a substantial amount of biodegradable solid waste, comprising various components such as the crown, peel, core, leaves, and stem. According to Tanamool et al. (2020), pineapple waste exhibits specific characteristics that are crucial for advancing the food, medicinal, and energy sectors (Tripathy et al., 2024). The viability of recycling pineapple leaf powder for removing heavy metals was investigated in a study conducted by Daochalermwong et al. (2020). The study explored the potential for a range of 4 to 5 recycling cycles. Therefore, the conversion of pineapple waste into a suitable adsorbent is of considerable importance, as it effectively addresses the problem of pollution caused by both inorganic and organic contaminants in various water sources, including groundwater, surface water, and drinking water. Aili Hamzah et al. (2021) conducted a recent study on the potential use of pineapple waste as a raw material for producing value-added products, including biogas, biofuels, biodegradable packaging, cellulose nanocrystals, and biosorbents.

Both fresh and processed pineapples generate substantial amounts of waste, including the stem, crown, core, peels, and leaves, which account for ∼50–60 % (*w*/w) of the total pineapple waste (Chen et al., 2021). Lack of proper handling of these wastes results in pollution of the environment through the release of leachate and landfill gas, as well as air pollutants such as furans, acid gases, dioxins, and particulate matter during burning (Ngoc-Dan Cao et al., 2022)(Salve & Ray, 2020). After cellulose, hemicellulose, and lignin, phytochemicals are the fourth significant pineapple byproduct and are a potential source for functional food components, such as phenolics, terpenes, and organic acids, which have inherent pesticidal properties that are natural antibacterial, antifungal, and antioxidants (Saini, 2023). The green technique of extracting high-value compounds from agriculture is one of the most effective ways to promote long-term global development.

The conversion of waste and by-products presents considerable potential and opportunity for fostering sustainable development by transforming them into valuable products (Li et al., 2022; Mohan et al., 2016; Pal et al., 2024; Sarangi et al., 2022). Harnessing these natural compounds not only offers a solution to managing food storage pests and diseases but also addresses the challenge of waste management in the pineapple industry. Research in this field has shown promising results, demonstrating the efficacy of pineapple waste extracts against a wide range of pests, including insects, fungi, bacteria, and viruses. These bioactives exhibit various modes of action, including repellency, disruption of insect feeding behavior, inhibition of fungal growth, and interference with bacterial reproduction, making them versatile candidates for pest and disease control (Awasthi et al., 2022).

Although there are works of literature on pineapple waste and its biological application (Fouda-Mbanga & Tywabi-Ngeva, 2022; Oliver-Simancas et al., 2024) Awasthi et al., 2022;(Vieira et al., 2022a) (Amores-Monge et al., 2022; Eixenberger et al., 2024; Sarangi et al., 2023; Tran et al., 2023) (George et al., 2023; Kumar & Singh, 2023; Meena et al., 2022; Mohd Ali et al., 2020; Nath et al., 2023; Sarangi et al., 2022), literature on the use of pineapple waste bioactives as a pesticide is lacking. Therefore, this review aims to consolidate existing knowledge on the pesticidal properties of pineapple waste-derived bioactives, assess their feasibility in controlling common food storage pests, and provide insights into their safety, environmental sustainability, and regulatory considerations in the last 5 years.

**Fig. 1:**
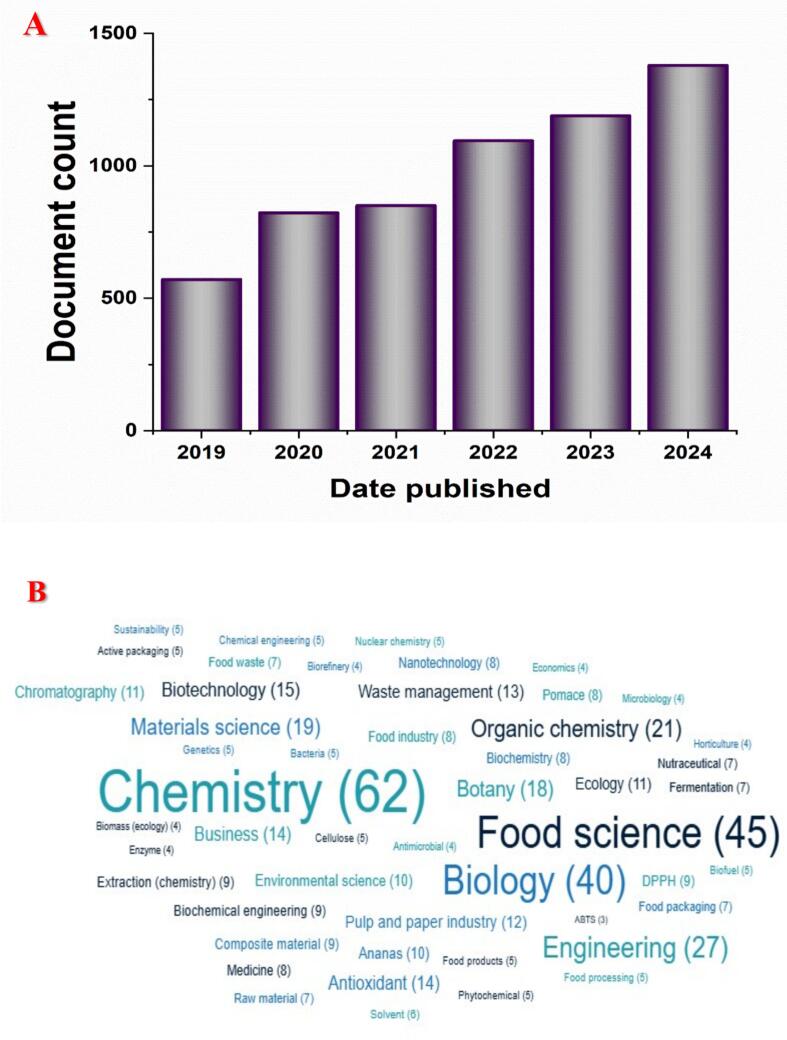
Scholarly works on pineapple by-products. (A) Number of publications from January 2019 to 31st December 2024 and (B) Fields of studies. (Data were taken from Lens.org).

The technique employed in this review adopts a thorough approach to conducting a literature evaluation, presenting a comprehensive overview of pineapple's bioactives for pesticide application over the last 5 years (Fig. 1). The data for this study were obtained from the Lens.org databases. The search terms employed were “pineapple,” “pineapple waste,” “pineapple byproducts,” “pineapple bioactive compounds,” “pineapple waste bioactive compounds,” “ pineapple bioactivities,” and “pineapple waste management.” These phrases were selected to encompass all pertinent papers on the subject (Fig. 1). The data were thoroughly reviewed, and relevant papers were chosen based on their relevance and quality. The eligibility criteria for this evaluation encompassed scholarly articles published in English up to December 31, 2024. Research on pineapple byproducts primarily originates from the fields of food science and chemistry (Fig. 1). By addressing these objectives, the review aims to contribute to the advancement of sustainable practices and the development of eco-friendly solutions for pest management in the food and agriculture sectors. (See Table 1.)

**Table 1:** Various Biopesticides and their pest's control.

Other biopesticide	Type of Entomopathogen	Target of pests	References
Bacterial Biopesticides	*Bacillus sphaericus, Bacillus lentimorbus, and Bacillus popilliae*	Larvae of Aedes spp., C*uliseta, Psorophora,* and Culex mosquitoes	(Falqueto et al., 2021)
	*Beauveria bassiana*	Whitefly	(McGuire & Northfield, 2020)
	*Bacillus popilliae*	Larvae of Japanese beetle	(Sabbahi et al., 2022a)
	*B. thuringiensis subsp. aizawai*	Wax moths	(Gounina-Allouane et al., 2022)
	*Bacillus thuringiensis subsp. kurstaki*	Moths	(Vimala Devi et al., 2020)
	*B. thuringiensis*	Elm Leaf Beetle, Alfalfa weevil	(Saberi et al., 2020)
Fungal Biopesticide	*Verticillium lecanii*	Nematodes, mites & thrips, scale insects, mealy bugs	(Pathania et al., 2022)
	*Verticillium lecanii*	Whitefy, cofee green bug. Homopteran pests	(Miranpuri & Khachatourians, 1995)
	*Trichoderma asperellum*	*Armillaria Fusarium Phytophthora Verticillium*	(Asad et al., 2014; De la Cruz et al., 2019)
	*Beauveria bassiana*	Cofee berry borer diamond back moth, thrips, grasshoppers	(Miranpuri & Khachatourians, 1995)
	*Hirsutella thompsonii*	Coleoptera, Diptera, Hemipter	(Sabbahi et al., 2022b)
	*Myrothecium verrucaria*	Nematodes	(Hagag, 2021)
	*Paecilomyces lilacinus*	Soil nematodes	(Moreno-Gavíra et al., 2020)
	*Hirsutella thompsonii*	Spider mites and whitefly	(Saranya et al., 2021)
	*Lagenidium giganteum*	Pest mosquito species	(Kaczmarek & Boguś, 2021)
	*Metarhizium brunneum*	Nematodes (pathogens)	(Hummadi et al., 2021)
	*Beauveria bassiana*	Whitefly	(McGuire & Northfield, 2020)
	*Paecilomyces fumosoroseus*	Insects and mealy bugs	(Abbas, 2020)
Nematodes Biopesticide Larvae of cabbage white butterfly	*Steinernema bicornutum, Heterorhabditis indica and H. bacteriophora.*	Leafminers	(Abbas, 2022)
	*Steinernema carpocapsae, Heterorhabditis bacteriophora, Steinernema feltiae, Steinernema carpocapsa.*		(Aioub et al., 2021)
	*Steinernema carpocapsae*	Potato tuber moth	(Ebrahimi et al., 2024)
Viral Biopesticide	*Potato tuber moth* GV (PhopGV)	*Phthorimaea operculella*	(Ayilara et al., 2023)
	*Nuclear polyhedrosis* virus (NPV)	Cotton bollworm Cotton budworm	(Chakraborty et al., 2023; Kaur et al., 2021)
	*Nucleopolyhedrovirus*	*Spodoptera exigua Lepidoptera*	(Chakraborty et al., 2023; Kaur et al., 2021)
	*Nucleopolyhedrovirus*	*Helicoverpa zea*	(Chakraborty et al., 2023; Kaur et al., 2021)
	*Granulovirus*	*Cydia pomonella*	(Chakraborty et al., 2023; Kaur et al., 2021)
	*Nucleopolyhedrovirus*	*Spodoptera litura*	(Chakraborty et al., 2023; Kaur et al., 2021)
	*Nucleopolyhedroviruses*	Lepidoptera	(Harish et al., 2021)
	*Imported cabbageworm* (PiraGV) NPV (AucaMNPV)	*Artogeia (Pieris) rapae*	(Ayilara et al., 2023)
Nanobiopesticides	Silver nanoparticles	*Xanthomonas axonopodis pv. citri, X. oryzae pv. oryzae and Ustilaginoidea virens*	(Roseline et al., 2019)
	Mesocyclops longisetus-derived nanoparticles	*Culex quinquefasciatus*	(Narware et al., 2019)
	Silver nanobiopesticide	*Alternaria solani, A. alternata*	(Narware et al., 2019)
	Mesocyclops scalpelliformis-derived nanoparticles	*Culex quinquefasciatus*	(Rodrigues et al., 2019)

## 2 Pineapple waste-derived compounds: Sources and composition

### 2.1 Pineapple waste and its composition

#### 2.1.1 Peel

**Fig. 2:**
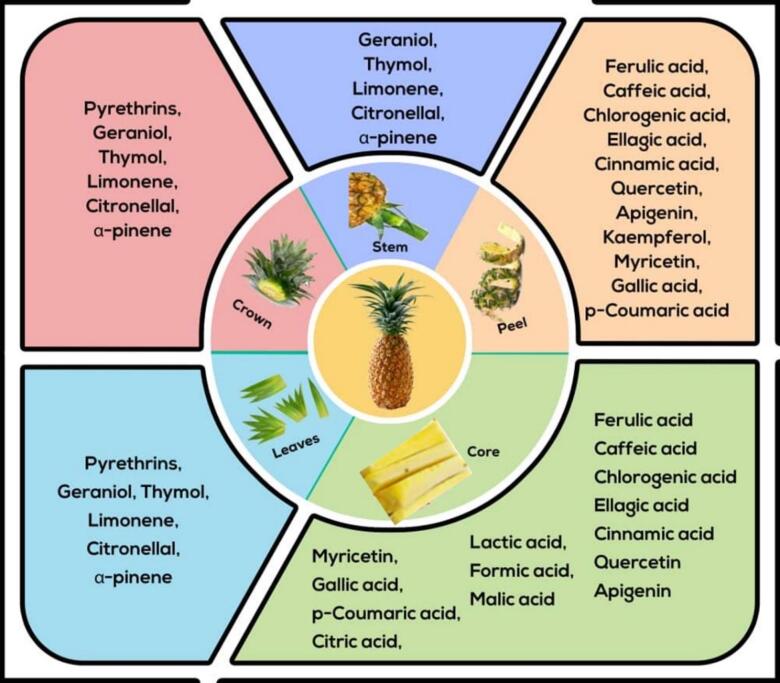
Pineapple waste and its derived bioactive compounds.

Peel residues, as depicted in Fig. 2, constitute a substantial proportion of the total waste generated during the processing of agricultural products, representing over 30 % of the overall byproducts. These residues are primarily composed of three key biopolymers: hemicellulose, cellulose, and lignin, with hemicellulose ranging from 6.5 to 35 g/100 g dry weight (d.w.), cellulose from 12 to 50 g/100 g d.w., and lignin from 5 to 30 g/100 g d.w. (Polania et al., 2023). Beyond their structural composition, peel residues have attracted significant scientific attention due to their abundant content of bioactive polyphenolic compounds such as salicylic acid, trans-cinnamic acid, myricetin, tannic acid, and p-coumaric acid, known for their potential health benefits and functional properties, making pineapple peels a promising resource for applications in the pharmaceutical, food, and cosmetic industries (Roda & Lambri, 2019).

Recent studies have further highlighted the practical applications of pineapple peel polyphenols (PPPs). For example, PPPs have demonstrated remarkable efficacy in combating soft rot in pakchoi (*Brassica chinensis* L.), a prevalent soil-borne disease that significantly hampers its production (Zhou & Tan, 2024). The study revealed that 4 mg/mL PPPs (referred to as the P1 treatment) led to substantial improvements in soil quality, reducing disease incidence by 79.97 %. Additionally, the P1 treatment enhanced the population of beneficial soil bacteria and increased soil enzyme activity. Notably, it also improved key growth parameters of pakchoi, including plant height (by 57.43 %), fresh weight (by 154.52 %), and chlorophyll content (by 50.11 %). These findings highlight the potential of pineapple peel polyphenols (PPPs) as a sustainable solution for improving soil health, reducing disease, and enhancing crop yields.

In another study, Wafaa et al. (2021) developed a zero-waste extraction process for pineapple peel polyphenol (PPP) powder on an industrial scale, utilizing solvent recovery and concentration techniques. This process also led to the creation of PPP antibacterial agents (PAAs) for environmental sterilization. The physicochemical and antibacterial properties of PAAs were thoroughly examined, and a comprehensive technical and financial analysis was conducted. The findings indicated that protocatechuic acid had the highest concentration (48.90 mg/L), followed by quercetin dihydrate (17.56 mg/L). The PAA sprays met all physicochemical property standards, including heat resistance and pH; at a concentration of 8 mg/mL, PAAs achieved 100 % bacterial inhibition at temperatures below 40 °C; and the production cost of 8 mg/mL PAA spray was economically viable, amounting to only $273.41 per ton at an industrial scale of 100,000 tons. These findings suggest that PAAs derived from pineapple peel residues hold significant promise for the developing renewable bacteriocins from fruit and vegetable waste materials, offering a sustainable and cost-effective alternative for industrial applications (Zhou et al., 2023).

#### 2.1.2 Crown

The conventional propagation of pineapples (*Ananas comosus*) predominantly relies on vegetative methods, with the crown being the most utilized structure. Additionally, other vegetative propagation techniques, such as the use of suckers and slips, are also employed. The pineapple crown (Fig. 2), which is typically removed during pineapple processing, accounts for approximately 25–30 % of the total waste generated in this industry.

Recent advancements in research have unveiled the untapped potential of pineapple crown as a functional ingredient, mainly due to its high antioxidant capacity. Studies have demonstrated that pineapple crown flour exhibits superior antioxidant activity compared to other conventional flours, positioning it as a promising candidate for the development of value-added food products. This enhanced antioxidant profile is primarily attributed to the presence of bioactive phenolic compounds, including ferulic acid, p-coumaric acid, and 4-hydroxybenzaldehyde, which are known for their health-promoting properties (Brito et al., 2021). These compounds not only contribute to the flour's bioactive potential but also underscore its nutritional significance.

#### 2.1.3 Core

The pineapple core (Fig. 2) constitutes approximately 15 % of the total pineapple waste, according to a study by Vieira et al. (2022b). Although often regarded as a byproduct and discarded, the pineapple core holds considerable potential for a wide range of industrial and culinary applications. For instance, it serves as a valuable raw material in the production of confectionery, alcoholic beverages, and vinegar, demonstrating its versatility in the food processing industry. Additionally, in traditional Asian culinary practices, the pineapple core is frequently incorporated into pickle-making, underscoring its cultural and gastronomic relevance (Abraham et al., 2023). This dual utility highlights the need to reconsider the core as more than mere waste but rather as a resource with significant economic and cultural value.

Beyond its practical applications, the pineapple core is distinguished by its rich nutritional and bioactive profile. It contains a high concentration of antioxidants, sugars, dietary fiber, and vitamin C, all of which contribute to its potential health-promoting properties. Furthermore, the core is a source of proteins, phenolic compounds, carotenoids, and flavonoids, which enhance its functional properties and make it a promising candidate for utilization in both the food and pharmaceutical industries. These bioactive components not only support human health but also offer opportunities for the development of nutraceuticals and functional foods.

#### 2.1.4 Stem

The stem of the pineapple plant (Fig. 2) is primarily composed of fibrous tissue, which plays a crucial role in maintaining the plant's structural stability. Despite its critical physiological functions, the pineapple stem is often regarded as a byproduct in agricultural practices due to its tough, fibrous texture and lower palatability compared to the fruit itself. As a result, its utilization in culinary applications remains minimal, and it is often overlooked in favor of the more commercially valuable fruit.

During the processing of pineapples, which includes harvesting, preparation, and packaging for either direct consumption or commercial distribution, the stem is typically discarded as agricultural waste. Research indicates that pineapple stems account for ∼2–5 % of the total waste generated during pineapple processing (Meena et al., 2022). This disposal of pineapple stems poses significant environmental and economic challenges. From an environmental perspective, the accumulation of such agricultural waste contributes to land pollution and represents a failure to manage resources efficiently.

Economically, the discarding of pineapple stems as waste represents a missed opportunity for value-added applications. The stem contains various bioactive compounds, including bromelain, phenolic compounds, and antioxidants, which have potential applications in the pharmaceutical, nutraceutical, and cosmetic industries. Additionally, the fibrous nature of the stem makes it a viable candidate to produce animal feed, biodegradable materials, or even as a substrate for bioenergy production. By neglecting to explore these potential applications, the agricultural and processing industries forfeit the opportunity to diversify revenue streams and reduce waste.

#### 2.1.5 Leaves

Pineapple leaves (Fig. 2) represent a valuable natural resource due to their rich composition of cellulose, lignin, hemicellulose, and various bioactives, including bromelain, flavonoids, and phenolic acids (Noel Lalhruaitluangi, 2024; Ramli et al., 2021; Wan Hashim et al., 2024). The cellulose content in pineapple leaves is particularly noteworthy, ranging between 60 % and 70 % of their dry weight. This high cellulose concentration underscores their potential as a sustainable and renewable raw material to produce cellulose-based products, such as bioplastics, paper, and textiles. Furthermore, the presence of lignin and hemicellulose in these leaves adds to their structural complexity, making them suitable for applications in bio-composite materials and bioenergy production.

In addition to their structural components, pineapple leaves are a significant source of diverse bioactives, which contribute to their wide-ranging applicability across various industries. These leaves are rich in phytochemicals, including phenolics, flavonoids, terpenes, and alkaloids, all of which are well-documented for their potent biological activities. The pesticidal properties of these bioactive compounds found in pineapple leaves highlight their potential as a natural alternative to synthetic pesticides in agricultural practices. This dual functionality, combining structural utility with bioactive benefits, positions pineapple leaves as a promising resource for both agricultural and pharmaceutical applications.

### 2.2 Overview of compounds extracted from pineapple waste

Numerous bioactive compounds can be found in pineapple waste, a byproduct of pineapple processing, as shown in Fig. 2. The several bioactive compounds isolated from pineapple waste and their effectiveness as environmentally benign pesticides for managing pests in food storage are thoroughly reviewed in this manuscript.

#### 2.2.1 Phenolic compounds

Phenolic compounds, also known as phenolics, represent a broad and structurally diverse class of secondary metabolites that are ubiquitously distributed throughout the plant kingdom (Polanía et al., 2023). These compounds are integral to plant physiology and are primarily localized within the cell walls and vacuoles of epidermal and subepidermal cells, where they fulfill a range of critical biological functions (Hikal et al., 2021). Certain phenolic compounds have been found to suppress the growth of competing weed species, further underscoring their ecological significance in plant survival and fitness (Hikal et al., 2021).

Pineapple waste has been recognized as a significant reservoir of bioactive phenolic compounds, which exhibit notable pesticidal properties. Among the key phenolic compounds identified in pineapple waste are ferulic acid, caffeic acid, chlorogenic acid, and cinnamic acid. These compounds have been extensively studied for their ability to disrupt essential physiological processes in insects, thereby serving as effective natural pesticides (Paz-Arteaga et al., 2024; Zampar et al., 2022). Specifically, these phenolics interfere with critical metabolic pathways by inhibiting enzymes that are crucial for chitin biosynthesis and detoxification, both of which are vital for insect survival (Anjali et al., 2023). Additionally, their potent antioxidant activity induces oxidative stress within pest cells, leading to cellular damage and ultimately resulting in mortality. In addition to phenolic compounds, pineapple waste is also rich in flavonoids, including quercetin, apigenin, kaempferol, and myricetin. These flavonoids contribute to pesticidal activity through multiple mechanisms. They disrupt ion balance, interfere with neurotransmitter signaling, and inhibit enzymatic activities that are essential for insect survival. Furthermore, these compounds exhibit antifeedant properties, which effectively deter pests from consuming stored food products, thereby providing an additional layer of protection against pest infestations (Paz-Arteaga et al., 2024).

Recent investigations have further expanded the understanding of pineapple waste's pesticidal potential by identifying the presence of other bioactives, including gallic acid, p-coumaric acid, and pyrethrins. These compounds have been shown to exhibit strong pesticidal activity through distinct mechanisms. Gallic acid, for instance, disrupts insect growth by interfering with hormonal regulation, while p-coumaric acid inhibits respiratory enzymes, resulting in reduced energy production and ultimately leading to pest mortality. Pyrethrins, on the other hand, act as potent neurotoxins that target the insect nervous system, causing paralysis and ultimately leading to death (Daraban et al., 2023; Isman, 2006; Rivera et al., 2023).

Phenolic compounds play a critical role in plant physiology, serving as both antioxidants and defense molecules against environmental stressors. Pineapple waste, as a rich source of these bioactive phenolics, offers significant potential for the development of sustainable pest management solutions. The diverse mechanisms of action exhibited by these compounds, ranging from enzymatic inhibition and oxidative stress induction to neurotoxicity, highlight their versatility and efficacy as natural pesticides. Given their multifaceted pesticidal properties, these compounds represent promising candidates for the formulation of eco-friendly pesticides that could minimize environmental impact while effectively controlling pest populations.

In conclusion, the bioactives present in pineapple waste, including phenolics, flavonoids, and other secondary metabolites, demonstrate significant potential for sustainable pest management. Further research into their applications, modes of action, and optimization for agricultural use could pave the way for the development of environmentally benign pesticides, contributing to more sustainable agricultural practices and reduced reliance on synthetic chemical pesticides.

#### 2.2.2 Terpenes

**Fig. 3:**
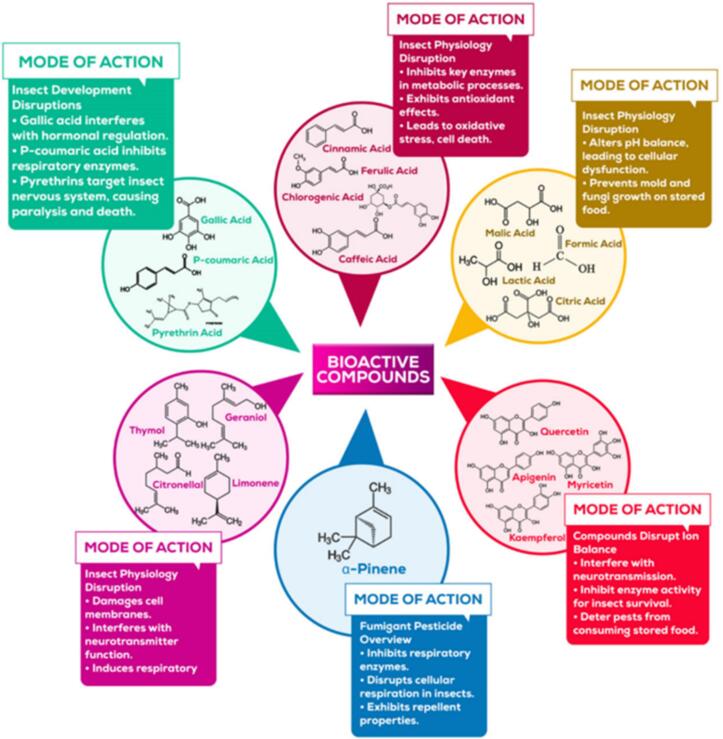
Bioactive compounds derived from Pineapple waste and its mode of action as biopesticide.

Terpenes constitute a remarkably diverse and extensive class of naturally occurring organic compounds, with an estimated 30,000 distinct polymeric structures identified to date. These compounds are fundamentally composed of isoprene units, chemically defined as 2-methyl-1,3-butadiene (CH₂C(CH₃)-CHCH₂), where the number of isoprene units (denoted as n) is typically two or greater (Attri et al., 2023). Terpenes play a multitude of ecological and physiological roles, ranging from regulating plant growth and development to serving as defense mechanisms against herbivorous insects and microbial pathogens. Additionally, they are known to disrupt cellular structures in target organisms, thereby contributing to their protective functions (Brito et al., 2021; Moreira et al., 2007). These roles are visually represented in Fig. 3 and systematically summarized in Table 2.

**Table 2:** Bioactive compounds and their pest control.

Bioactive compounds	Target pests	Mode of action	References
	*Staphylococcus aureus*, *Pseudomonas aeruginosa,* and *Escherichia coli*	Growth inhibition due to the C1 hydroxyl group of the monoterpene nucleus.	(Boye et al., 2020)
	*Staphylococcus aureus, Escherichia coli*	Cellular membrane disruption	(Ferreira et al., 2019)
	*Escherichia coli, Listeria monocytogenes,* and *Staphylococcus aureus*	Alteration of the membrane of the bacteria	(Kim et al., 2020)
Thymol	*Leuconostoc citreum*	Disruption of cell membrane resulting in the leakage of intracellular constituents	(Lee et al., 2020)
	*Rhipicephalus sanguineus*	Changes in the morphology and calcium levels in the cells and tissues of the singanglia and salivary glands	(Matos, Daemon, et al., 2019)
	*Solenopsis invicta Buren, Solenopsis richteri Forel, Solenopsis invicta × Solenopsis richteri*	Repellants	(Paudel et al., 2023)
	*Fusarium graminearum and deoxynivalenol*	Caused hyphal break and shrink, damaged the structures of cell membrane, cell wall, vacuoles and organelles in the hypha.	(Jian et al., 2023)
	*Aspergillus* spp	Inhibitory or enhancing effect on microbial growth	(Bulkan et al., 2022)
Limonene	*Aspergillus flavus and Aflatoxins*	Inhibition of fungal growth	(Li et al., 2021)
	*Fusarium graminearum*	Inhibition of fungal growth	(Luchesi et al., 2022)
	*Aspergillus flavus*	Inhibits fungal growth and toxin synthesis	(Xiang et al., 2020)
	*Staphylococcus aureus*	Inhibit Coa activity	(Wang, Li, et al., 2019)
	*Enterococcus faecalis*	Disturbing glycolytic, protein translation-elongation, and protein folding pathways	(Qayyum et al., 2019)
Quercitin	*Staphylococcus aureus*	Inhibit the formation of recalcitrant biofilms of *S. aureus*	(Lee et al., 2013)
	*Deoxynivalenol*	Inhibition of the mitochondrial apoptosis pathway and lipid peroxidation	(Pomothy et al., 2021)
	*Spodoptera frugiperda*	Repellants	(Herrera-Mayorga et al., 2022)
	*Spodoptera frugiperda*	Growth inhibition	(Herrera-Mayorga et al., 2022)
	*armyworm Mythimna separata*	Decreased the susceptibility to CGA in resistance strain, indicating three CarE genes play crucial roles in CGA detoxification	(Lin et al., 2023)
	*Bemisia tabaci*	Paralysis and intoxication by inhibiting herbivore phosphodiesterase activity	(Wang et al., 2023)
chlorogenic acid	*Mythimna separata (Walker)*	Affects insect feeding behavior, growth, development, and reproduction, and they may have lethal effects on specific insects.	(Lin et al., 2022)
	*Sweetpotato weevil*	Inhibits sweetpotato weevil attack	(Liao et al., 2020)
	*Aphis gossypii Glover*	Inhibit growth	(Elmasry et al., 2020)
	*Spodoptera frugiperda*	Acetyl- cholinesterase inhibition	(Rodríguez-Cervantes et al., 2024)
	*Staphylococcus aureus, Pseudomonas aeruginosa*	Exhibited strong antioxidant activity based on DPPH radical scavenging capacity	(Taiwo et al., 2019)
	*Leishmania amazonensis*	Inhibition of growth	(Araújo et al., 2019)
Kaempferol	*Trypanosoma brucei, Leishmania mexicana*	Inhibition of growth	(Alotaibi et al., 2021)
	*Trypanosoma* spp.*, Plasmodium* spp.	Inhibition of growth	(Abdallah et al., 2021a; Abdallah et al., 2021b)
caffeic acid	*Tribolium confusum, Cryptolestes ferrugineus, Trogoderma granarium*	Preventing insects from feeding or demonstrating repellent and growth inhibition effects	(Zarmakoupi et al., 2023)
	*Spodoptera litura*	Growth inhibition	(Punia et al., 2023)
Myricetin	*Sitophylus oryzae*	Inhibition of growth	(M'hamdi et al., 2024)
	*Tribolium castaneum Hebst*	Repellent	(Buxton et al., 2020)
	*Aedes aegypti L*	Impair the growth	(Araújo et al., 2021)
Cinnamic Acid	*Plutella xylostella L*	Impair the growth	(Huang et al., 2025)
	*Aedes aegypti*	Inhibition of growth	(Bezerra França et al., 2021)
	*Sclerotinia sclerotiorum*	Impair the growth	(Wang, Sun, et al., 2019)
	*Botrytis cinerea*	Delay in fungal growth by influencing the spore germination and the growth rate of the mycelium	(Patzke & Schieber, 2018)
	*Xanthomonas oryzae pv. oryzae (Xoo), tobacco mosaic virus*	Disrupt the three-dimensional structure of the TMV coat protein, making TMV particles unable to self-assemble	(Dai et al., 2022)
ferulic acid	*Fusarium verticillioides, Rhizoctonia solani, Aphis gossypii*	Decreased the daily reproductive rate, reproductive period, and mean survival percentage	(Khamis et al., 2023)
	*Tribolium castaneum*	Impair the growth	(Ghada et al., 2025)
	*podoptera litura F*	Impair the growth of *S. litura* larvae	(Punia et al., 2021)
Gallic acid	*Aphis craccivora Koch*	Impair the growth	(Dolma et al., 2022)
	*Ectropis obliqua*	Triggers a direct defense response against tea geometrid larvae	(Zhang et al., 2022)
	*Tribolium castaneum, Helopeltis antonii, Ephestia cautella, Bemisia tabaci*	Affects acetylcholine, γ-aminobutyric acid, and octopaminergic receptors as well as respiratory pathways.	(Istianto & Emilda, 2021)
	*Ulomoides dermestoides*	Affects acetylcholine, γ-aminobutyric acid, and octopaminergic receptors as well as respiratory pathways.	(Plata-Rueda et al., 2020)
citronellal	*Spodoptera frugiperda*	Impair the growth	(Firdausiah et al., 2023)
	*stink bugs, brown planthoppers, grasshoppers, ladybugs, and aphids*	Natural repellents	(Mahmud et al., 2022)
	*Staphylococcus and Escherichia*	Impair the growth	(Venancio et al., 2024)
	*Sitophilus zeamais*	Natural repellents	(Langsi et al., 2020)
α-pinene	*Myzus persicae*	Inhibiting growth and repellents.	(Chohan et al., 2023)
	*Sitophilus zeamais*	Inhibit growth	(de Carvalho Brito et al., 2024)

One notable source of bioactive terpenes is pineapple waste, which contains a variety of terpenoid compounds, such as geraniol, thymol, limonene, and citronellal. These volatile organic compounds exhibit significant pesticidal properties, making them valuable in managing insect populations. The pesticidal mechanisms of these terpenes involve inducing physiological disruptions in insects, including damage to cellular membranes, interference with neurotransmitter activity, and the induction of respiratory toxicity (Qasim et al., 2024). Furthermore, their potent aromatic characteristics enhance their efficacy as natural pest deterrents, thereby preventing insect infestations and protecting stored food products from contamination.

Among the terpenes found in pineapple waste, α-pinene is particularly noteworthy for its role as a fumigant pesticide. This compound exerts its pesticidal effects by inhibiting key respiratory enzymes and disrupting cellular respiration in insects. In addition to its direct toxic effects, α-pinene also exhibits strong repellent properties, which effectively deter insect infiltration in storage environments. This dual functionality not only mitigates the risk of food contamination but also underscores the potential of terpenes as sustainable alternatives to synthetic pesticides. The application of terpenes in post-harvest storage and food preservation represents a promising avenue for developing eco-friendly pest control strategies, aligning with global efforts to reduce reliance on chemical pesticides and promote environmental sustainability.

In summary, terpenes are a versatile and ecologically significant class of compounds with a wide range of applications in plant defense, pest management, and food preservation. Their structural diversity, coupled with their bioactive properties, positions them as valuable tools in the development of sustainable agricultural practices and integrated pest management systems. Further research into the extraction, characterization, and application of terpenes from agricultural waste, such as pineapple byproducts, could yield innovative solutions for addressing challenges in food security and environmental conservation.

#### 2.2.3 Organic acids

Organic acids represent a diverse and functionally significant group of acidic organic compounds, classified according to the number of carboxyl groups they contain. These compounds are widely utilized across various industries, including bakery, dairy, livestock feed, beverages, cosmetics, and pharmaceuticals, due to their versatile chemical properties and biological activities. The growing market demand for organic acids, driven by an increasing preference for natural and sustainable ingredients, has spurred significant advancements in their production methods and applications. Among the innovative approaches to organic acid production, the utilization of agricultural waste has emerged as a promising and sustainable strategy. Specifically, pineapple waste, which is abundant in fermentable sugars, has been identified as an optimal substrate for microbial fermentation. This process enables the efficient and cost-effective generation of various organic acids, thereby contributing to both waste valorization and the production of valuable chemical compounds (Aili Hamzah et al., 2021).

Recent studies have highlighted the potential of organic acids derived from pineapple waste, such as citric acid, lactic acid, formic acid, and malic acid, in exhibiting pesticidal properties (Ismail et al., 2024). These organic acids exert their pesticidal effects primarily through their acidic nature, which disrupts the physiological and biochemical processes of target insects. By altering the pH balance within the insect's body, these compounds induce cellular dysfunction, leading to impaired metabolic activities and, ultimately, mortality. This mode of action not only underscores the efficacy of organic acids as natural pesticides but also positions them as environmentally benign alternatives to synthetic chemical pesticides. The use of organic acids for pest management aligns with the principles of sustainable agriculture, as it offers a dual benefit: reducing reliance on harmful synthetic chemicals and addressing the challenge of agricultural waste disposal.

The integration of organic acids into pest management strategies represents a significant step toward achieving sustainable agricultural practices. By leveraging the pesticidal properties of these compounds, it is possible to develop eco-friendly solutions that mitigate the environmental impact of conventional pesticides while promoting the efficient use of agricultural by-products. Furthermore, the production of organic acids from pineapple waste exemplifies the potential of circular economy principles, wherein waste materials are transformed into valuable resources. This approach not only enhances resource efficiency but also contributes to reducing environmental pollution associated with agricultural waste accumulation.

In conclusion, organic acids derived from pineapple waste possess immense potential as sustainable and multifunctional compounds with applications that span industrial production and agricultural pest management. Their ability to serve as natural pesticides, coupled with their role in addressing agrarian waste challenges, underscores their importance in advancing sustainable development goals. Future research should focus on optimizing production processes, exploring additional applications, and assessing the long-term environmental and economic implications of utilizing organic acids across various sectors.

## 3 Pineapple waste-derived bioactive compounds as pesticides and mechanism of action

### 3.1 Bioactive compounds as biopesticides

There has been a significant increase in studies focused on understanding the complex chemical compositions seen in different plant groups in recent years. The rise in interest is driven by a growing desire to utilize the extensive capabilities of natural plant products in various fields, including medicine, agriculture, and cuisine (Ristaino et al., 2021). At the core of this growing topic is the increasing investigation of botanical pesticides, indicating a promising transition toward sustainable pest control methods. *Tanacetum cinerariifolium* is rich in bioactive compounds, including phenolics, esters, and flavonoids, and is known for its potent ability to repel pests (Lengai et al., 2020). Similarly, the foliage of *Mentha piperita*, commonly known as peppermint, contains a wide range of chemical constituents, including tannins and flavonoids. These chemicals collectively have a significant role in the plant's effectiveness in managing pests and controlling diseases. As scientists explore the chemical complexities of various plant species, they are uncovering their exact compositions and the biological impacts of these compounds. This knowledge enables the development of innovative solutions derived from plants to address pressing issues in agriculture, medicine, and other fields. This interdisciplinary undertaking not only enhances our comprehension of natural systems but also presents significant potential for promoting sustainable innovation and prudent resource management. By harnessing the untapped potential of botanical chemicals, we can establish a more harmonious and mutually beneficial relationship with the natural world while effectively addressing pressing societal needs.

To promote the upcycling of these by-products, Moreira et al. characterized the phenolic profile of hydroethanolic extracts obtained from pineapple peel and crown leaves and evaluated their in vitro bioactivity. The HPLC-DAD-ESI/MS analysis allowed the identification of 25 phenolic compounds, including phenolic acids and flavonoids. The antioxidant, cytotoxic, and antimicrobial activity assays highlighted the peel extract as the most promising and, therefore, it was incorporated into a traditional Portuguese pastry cake as a functional ingredient. The nutritional parameters of the developed food were not affected by the incorporation of the extract; however, it promoted antioxidant activity during its shelf life. Overall, the pineapple peel and crown appear to be promising by-products that the food industry can exploit through a circular economy approach (Moreira et al., 2022)**.** Furthermore, byproducts contain a high concentration of health-promoting phytochemicals. The phenolic profile of the pineapple crown and its essential oils were determined using UPLC-ESI-QTOF-MS and GC-FID/MS, respectively. Antimicrobial activity was tested against five bacteria, and antioxidant activity was assessed. 177 phenolics were detected in byproducts (ferulic acid, p-coumaric acid, and 4-hydroxybenzaldehyde) and 56 in cabbage-stalk flour (CSF) (quercetin 3-O-glucuronide, chlorogenic acid, and p-coumaric acid). Pineapple-crown flour (PCF) has a unique profile of stilbenes, lignans, and antioxidant capacity, particularly in its bound extracts. Fatty acids, esters, and terpenes were the most common volatile chemicals in CSF (30 fatty acids) and PCF (41 esters and terpenes). A thorough metabolomic analysis revealed CSF and PCF as a possible source of PC, with potent antioxidant and distinct antibacterial activity.

#### 3.1.1 Mechanisms of action of bioactive compounds

Biopesticides contain bioactives that target various types of pests, including insects, fungi, bacteria, nematodes, and plant cells infected with viruses (Fig. 3). These mechanisms include qualities that repel pests, effects that impede their activity, protein denaturation, and other processes that impair their ability to function. The specific botanical ingredient and the targeted pest determine the effectiveness of these mechanisms.

For efficient pest management techniques, it is necessary to have a full understanding of the mode of action, which involves the physical, biological, and chemical interactions between the insect and the pesticide (Tadesse Mawcha et al., 2025). The following sections will outline the many mechanisms by which biopesticides function, with a specific focus on targeting specific types of agricultural pests.

### 3.2 Mode of action of bioactive compounds in the pineapple waste against insect pests

Pineapple waste, a byproduct of the pineapple industry, has garnered significant attention due to its rich content of bioactives, which exhibit a wide range of effects on insect pests. As illustrated in Table 2 and Fig. 3, these compounds exert multiple effects on insects, including repellency, deterrence of feeding and oviposition, toxicity, mortality, and disruption of physiological processes. This multifaceted activity makes pineapple waste a promising candidate for the development of natural insecticides, offering a sustainable alternative to synthetic chemical pesticides.

Phenolic compounds are ubiquitously distributed in plants and are known for their antioxidant, anti-inflammatory, and antimicrobial properties. In pineapple byproducts, phenolic compounds such as flavonoids, phenolic acids (e.g., ferulic acid), and tannins are particularly abundant. These compounds have been demonstrated to possess insecticidal properties, functioning as feeding deterrents or toxic agents against various insect pests (Zhu et al., 2023). The mechanisms through which phenolic compounds exert their effects are multifaceted. They can interfere with the digestive processes of insects by disrupting enzyme functions or inducing oxidative stress, ultimately leading to mortality. For instance, phenolic acids and tannins have been shown to inhibit digestive enzymes in stored-product pests, resulting in stunted growth or death (Singh et al., 2021). Additionally, these compounds can render food sources less palatable, causing insects to abandon them, thereby reducing feeding damage (War et al., 2020).

Pineapple byproducts are also rich in organic acids, including citric acid, malic acid, and acetic acid, which are primarily concentrated in the pineapple peel. These acids play crucial roles in various biochemical processes within the plant and have been found to influence the metabolic processes of insects. Organic acids can disrupt the gut microbiota of insects or act as direct toxicants, leading to reduced survival rates. One of the primary mechanisms by which organic acids exert their effects is by altering the pH balance within the insect's digestive system. This pH disruption can impair nutrient absorption and microbial balance, contributing to insect mortality. Furthermore, the sour taste of these acids may render treated food sources less attractive to pests, further deterring feeding.

Terpenes, including limonene and α-pinene, are another class of bioactives found in pineapple byproducts. These compounds are known for their pungent odors and have been shown to repel or even kill certain insect species. Terpenes can affect the nervous systems of insects, disrupting their ability to locate food or mates. In some cases, terpenes act as neurotoxins, disturbing nerve signals and leading to paralysis or death (Boncan et al., 2020). Additionally, terpenes can interfere with insect respiration or feeding behavior, causing behavioral changes that ultimately lead to reduced pest populations (Troczka et al., 2021). The repellent properties of terpenes make them particularly valuable in integrated pest management strategies, as they can prevent insects from infesting treated areas or food sources.

One of the primary mechanisms by which pineapple byproducts deter insect pests is through feeding deterrence. The phenolic compounds present in pineapple byproducts, particularly flavonoids and tannins, have been shown to disrupt the feeding habits of various pests. These compounds can block digestive enzymes or make food sources less palatable, causing insects to abandon them (War et al., 2020). In addition to phenolic compounds, the sour taste of organic acids may contribute to the unattractiveness of treated food sources. Insects that consume extracts or compounds derived from pineapple byproducts often experience a breakdown in their digestive systems due to the inhibitory effects of phenolic acids and organic acids on digestive enzymes. This disruption can lead to an inability to process food, resulting in stunted growth or death.

Pineapple byproducts, rich in phenolic compounds, organic acids, and terpenes, have demonstrated significant potential as natural insecticides. The bioactives in these byproducts act through multiple mechanisms, including feeding deterrence, digestive disruption, repellency, and toxicity, all of which contribute to reducing pest populations and minimizing damage to food stores. As the global agricultural sector increasingly seeks sustainable practices, the utilization of pineapple byproducts as natural pesticides offers an eco-friendly alternative to synthetic chemical insecticides. Further research and development of pineapple byproduct-based formulations could provide a valuable addition to integrated pest management strategies, promoting both environmental sustainability and agricultural productivity.

### 3.3 Mode of action of bioactive compounds in the pineapple waste against fungal pathogens

Biopesticides are composed of a diverse array of secondary metabolites, including terpenes, phenols, alcohols, alkaloids, tannins, and other bioactives. These metabolites exhibit significant toxicity toward fungi by targeting critical cellular structures and functions, thereby inhibiting the growth and proliferation of various pests, as illustrated in Table 2. Among the natural sources of these bioactives, pineapple byproducts, such as the peel, core, and crown, are particularly noteworthy due to their rich content of phenolics, organic acids, and terpenes, which demonstrate potent antimicrobial properties. These compounds offer a promising alternative to synthetic fungicides, aligning with the growing demand for sustainable and eco-friendly solutions in food preservation and agricultural practices.

Pineapple byproducts are abundant in phenolic compounds, including flavonoids and phenolic acids, which are widely recognized for their antioxidant and antimicrobial activities. These compounds play a crucial role in combating fungal infections by disrupting the integrity of the fungal cell wall and modulating enzyme activities. For instance, ferulic acid, a phenolic acid found in pineapple byproducts, has been shown to exhibit antifungal effects by interfering with the structural integrity of fungal cell walls and inhibiting key enzymatic processes in pathogenic fungi (Suriyaprom et al., 2022). Additionally, research has shown that pineapple peel extracts, rich in phenolic compounds, can effectively inhibit the growth of various fungal species, highlighting their potential as natural antifungal agents (Ortega-Hernández et al., 2023).

In addition to phenolics, pineapple byproducts are a significant source of organic acids, such as citric acid and acetic acid. These compounds exert antifungal effects by lowering the pH of the environment, creating conditions that are inhospitable for fungal growth. Citric acid, in particular, has been shown to inhibit the growth of foodborne fungal pathogens, including *Aspergillus niger* and *Penicillium* spp. (Matos, Cardoso, et al., 2019). Organic acids also disrupt the membrane integrity of fungal cells, impairing their metabolic processes and growth. This dual mechanism of action, involving environmental acidification and membrane disruption, makes organic acids highly effective in controlling fungal proliferation.

Furthermore, pineapple byproducts contain terpenes, including monoterpenes and sesquiterpenes, which are volatile compounds known for their potent antifungal activity. Notable examples include limonene and β-caryophyllene, which have been shown to inhibit fungal growth and spore germination (Masyita et al., 2022). These terpenes interact with fungal cell membranes, causing structural disruptions that lead to the leakage of intracellular contents and, ultimately, cell death. The multifaceted antifungal properties of these compounds make them valuable candidates for the development of natural fungicides.

The bioactives in pineapple byproducts target various aspects of fungal cell physiology and biochemistry, making them highly effective against a wide range of fungal pathogens. Phenolic compounds and organic acids primarily compromise the integrity of the fungal cell wall, a critical structure that maintains cell shape and function. By interfering with cell wall biosynthesis or causing physical damage, these compounds render fungi more susceptible to osmotic stress and environmental damage, ultimately leading to cell death (Bertan et al., 2022).

Terpenes and organic acids also disrupt the integrity of fungal cell membranes. This disruption increases membrane permeability, leading to the leakage of essential cellular components, including ions, enzymes, and proteins. For example, limonene interacts with the lipid bilayer of fungal cell membranes, inducing structural changes that increase permeability and ultimately lead to cell death. Additionally, phenolic compounds inhibit key enzymes involved in fungal cell wall synthesis, such as chitinase and glucanase. By blocking these enzymes, phenolics prevent the proper formation and repair of the fungal cell wall, thereby hindering the pathogen's ability to grow and multiply.

Organic acids, such as citric acid, not only lower the pH of the environment but also enhance the antifungal efficacy of phenolic compounds. The combination of low pH and the antimicrobial properties of organic acids disrupts fungal metabolism and protein synthesis, further inhibiting fungal growth (Matos, Cardoso, et al., 2019). This synergistic effect underscores the potential of pineapple byproducts as a source of multifunctional antifungal agents.

The bioactives derived from pineapple byproducts hold significant promise for applications in food preservation. As concerns over the use of synthetic chemicals in food production continue to grow, there is an increasing demand for natural preservatives that are both effective and environmentally sustainable. Pineapple byproducts can be utilized to develop eco-friendly anti-fungal agents that extend the shelf life of food products while maintaining their safety and quality. For instance, pineapple-based antifungals could be incorporated into food packaging materials or applied as coatings for fresh produce to prevent fungal contamination and spoilage.

Pineapple byproducts, particularly their phenolic compounds, organic acids, and terpenes, exhibit remarkable potential as natural antifungal agents. Their mechanisms of action—ranging from cell wall disruption and membrane damage to enzyme inhibition and environmental acidification make them effective against a variety of foodborne fungal pathogens. With further research and development, these bioactives could be harnessed as sustainable and safe alternatives to conventional chemical fungicides in the food industry. This would not only enhance food safety but also contribute to reducing the environmental impact associated with synthetic preservatives. The utilization of pineapple byproducts as a source of biopesticides represents a significant step toward achieving sustainable agricultural and food preservation practices.

### 3.4 Mode of action of bioactive compounds in the pineapple waste against bacterial pathogens

Phenolic compounds, such as flavonoids, tannins, and phenolic acids (e.g., gallic acid, caffeic acid, and ferulic acid), are abundantly present in pineapple byproducts. These compounds are well-known for their antioxidant and antimicrobial activities, which are primarily attributed to their ability to disrupt bacterial cell membranes and interfere with cellular processes (Zeng et al., 2023). For instance, gallic acid has demonstrated efficacy in inhibiting the growth of pathogenic bacteria such as *Escherichia coli* and *Staphylococcus aureus*. Its mechanism of action involves damaging bacterial cell walls and inducing oxidative stress, which compromises bacterial viability (Chávez-González et al., 2020).

Phenolic compounds exert their antimicrobial effects through multiple pathways. They can interact with bacterial cell membranes, causing structural damage and increasing membrane permeability. Additionally, tannins, a subclass of phenolic compounds, bind to bacterial membrane proteins, disrupting their function and integrity (Chávez-González et al., 2020).

Furthermore, phenolic compounds generate reactive oxygen species (ROS) within bacterial cells, causing oxidative damage to DNA, proteins, and lipids. This oxidative stress overwhelms the bacterial antioxidant defense systems, ultimately resulting in cell death (Gómez-García et al., 2021).

Pineapple byproducts are also rich in organic acids, including citric acid, malic acid, and ascorbic acid. These compounds contribute to antimicrobial activity by lowering the pH of the environment, creating unfavorable conditions for bacterial growth. Organic acids can penetrate bacterial cells and disrupt intracellular pH homeostasis, leading to cell death (Gómez-García et al., 2021). For example, citric acid has been shown to inhibit the growth of *Salmonella* spp. by chelating essential metal ions required for bacterial enzyme activity, thereby disrupting metabolic processes (Peh et al., 2020). Moreover, organic acids such as citric acid can inhibit key bacterial enzymes involved in energy production, including ATP synthase, further compromising bacterial survival (Peh et al., 2020).

Terpenes, such as limonene and β-caryophyllene, are volatile compounds found in pineapple byproducts that exhibit potent antimicrobial activity. These compounds disrupt bacterial cell membranes and inhibit biofilm formation, making them effective against a range of foodborne pathogens. For instance, limonene has been shown to increase membrane permeability in *Listeria monocytogenes*, causing the leakage of cellular contents and leading to cell death (El Fannassi et al., 2023). Terpenes also interfere with bacterial quorum sensing and adhesion mechanisms, reducing the ability of pathogens such as *Pseudomonas aeruginosa* to form biofilms and resist antimicrobial agents (Thongphichai et al., 2023).

The combined action of phenolic compounds, organic acids, and terpenes enhances their antimicrobial efficacy. These bioactives target multiple cellular processes, including cell membrane integrity, oxidative stress response, enzyme activity, and biofilm formation. For example, phenolic compounds and terpenes can synergistically disrupt bacterial cell membranes, while organic acids exacerbate intracellular damage by altering pH homeostasis and inhibiting metabolic enzymes. This multi-target approach makes it difficult for bacteria to develop resistance, highlighting the potential of these compounds as natural antimicrobial agents.

The bioactives derived from pineapple byproducts have significant potential for use as natural preservatives in the food industry. They can be incorporated into food packaging materials, coatings, or directly added to food products to extend shelf life and prevent contamination by foodborne pathogens. For instance, pineapple peel extracts have been used to develop antimicrobial films that effectively inhibit the growth of aerobic mesophilic and *Pseudomonas* spp in meat products (Lourenço et al, 2020). Such applications not only enhance food safety but also contribute to reducing food waste by utilizing byproducts that would otherwise be discarded.

Pineapple byproducts are a valuable source of bioactives with potent antimicrobial properties. Phenolic compounds, organic acids, and terpenes derived from these byproducts exhibit multiple mechanisms of action against foodborne pathogens, including cell membrane disruption, oxidative stress induction, enzyme inhibition, and biofilm inhibition. Harnessing these compounds for food safety applications offers a natural and sustainable alternative to synthetic preservatives. However, further research is needed to optimize extraction methods, evaluate the safety and efficacy of these compounds in real-world food systems, and explore their potential in other industries, such as pharmaceuticals and cosmetics. By leveraging the antimicrobial potential of pineapple byproducts, we can address both food safety challenges and sustainability goals.

### 3.5 Mode of action of bioactive compounds in the pineapple waste against Virus Pathogens

Pineapple byproducts, particularly the peels and cores, are rich sources of phenolic compounds, which are secondary metabolites with significant bioactive properties. These compounds, including flavonoids, phenolic acids, and tannins, are well-documented for their antioxidant and antiviral activities. Notable phenolic acids, such as ferulic acid, caffeic acid, and gallic acid, which are abundant in pineapple byproducts, have demonstrated potent antiviral effects. These phenolics exert their antiviral mechanisms by disrupting viral replication, inhibiting viral attachment to host cells, and inactivating viral particles through oxidative processes (Moreira et al., 2022). In addition to phenolic compounds, pineapple byproducts contain a variety of organic acids, including citric acid, malic acid, and ascorbic acid. These acids contribute to the fruit's characteristic acidity and possess antimicrobial and antiviral properties. By lowering the pH of the environment, organic acids create unfavorable conditions for viral survival. Furthermore, they can disrupt the structural integrity of viral envelopes and capsid proteins, rendering the virus non-infectious (Górniak et al., 2019).

Another group of bioactives found in pineapple byproducts is terpenes, which include limonene and β-caryophyllene. These volatile compounds are known for their antimicrobial and antiviral activities. Terpenes interact with viral membranes, causing structural damage and inhibiting viral entry into host cells. For instance, limonene has been reported to exhibit virucidal activity against enveloped viruses by destabilizing their lipid membranes (Moreira et al., 2022). The antiviral properties of these bioactives are further enhanced by their ability to directly interact with viral particles, leading to structural damage. Phenolic compounds and terpenes can bind to viral capsid proteins, inducing conformational changes that prevent the virus from attaching to host cells. Similarly, organic acids can denature viral proteins and nucleic acids, thereby inactivating the virus (Moreira et al., 2022).

Phenolic compounds also play a critical role in inhibiting viral replication by targeting key enzymes such as RNA-dependent RNA polymerase and proteases. By blocking these enzymes, phenolics prevent the synthesis of viral RNA and proteins, effectively halting the viral life cycle (Górniak et al., 2019). Additionally, certain bioactives, particularly phenolics, can modulate the host immune response. They enhance the production of antiviral cytokines and stimulate the activity of immune cells, thereby providing an indirect mechanism to combat viral infections more effectively (Moreira et al., 2022).

The virucidal properties of bioactives derived from pineapple byproducts hold significant promise for applications in food safety. These compounds can be integrated into food packaging materials, surface sanitizers, and washing solutions to mitigate the risk of viral contamination. For example, pineapple peel extracts have been tested as natural sanitizers for fresh produce, demonstrating efficacy against foodborne viruses without compromising the quality of the food (Banerjee et al., 2018).

## 4 Other uses of pineapple waste-derived bioactive compounds

The contamination of water resources by dyes, heavy metals, pesticides, inorganic and organic pollutants, primarily due to municipal effluents, industrial discharges, and agricultural activities, represents a significant global environmental challenge (Sarangi et al., 2022; Solangi et al., 2021). Addressing this issue requires the development of sustainable, cost-effective, and efficient adsorbents for wastewater treatment. In this context, pineapple by-products have emerged as a promising candidate to produce low-cost and eco-friendly adsorbents. The inherent presence of lignin, hemicellulose, and cellulose, along with reactive functional groups such as carbonyl, carboxyl, and hydroxyl group, makes pineapple by-products a suitable and sustainable material for adsorbent production (Tran et al., 2023).

Pineapple by-products can be utilized either in their unprocessed form or subjected to physical and chemical modifications to enhance their adsorption capacity. Physical modifications include drying, grinding, and heat treatment, while chemical modifications encompass processes such as graft copolymerization, disulfide treatment, saponification, deamination, pyrolysis, and protonation (Nyika & Dinka, 2023). For instance, Abd Ghapar et al. (2020) demonstrated the efficacy of chemically modified pineapple peels, specifically through zinc chloride (ZnCl₂) impregnation, which removed pigments and acid-soluble oligosaccharides, resulting in a porous structure. This modification achieved a 55.26 % removal efficiency of Fe(III) ions, highlighting a dose-dependent adsorption trend.

Recent studies have further explored the potential of pineapple by-products in wastewater treatment. Nerdy et al. (2023) extracted pectin from pineapple peels and utilized it as an adsorbent, achieving removal efficiencies of 64.8 % to 72.4 % for heavy metals (cadmium and lead) and dyes (Malachite Green and Congo Red). The adsorption mechanism was attributed not only to the porous surface structure but also to the formation of pectin hydrogels, which facilitated hydrogen bonding between contaminants and pectin. Additionally, the binding of divalent ions with carboxyl groups in pectin led to the formation of water-insoluble crosslinks, known as “egg-box” structures, which enhanced rapid gelation. The metal-binding capacity of pectin was found to be influenced by its structural properties, particularly the degree of methylesterification.

In another study, Dai et al. (2020) processed pineapple peel waste through water, alkali, bleaching, and combined bleaching/alkali treatments to selectively remove lignin and hemicellulose. The treated pineapple peel was then converted into hydrogels (WT-PPH, AT-PPH, BTPPH, and PPCH) using a dissolution-regeneration process in ionic liquid. The adsorption of Congo Red (CR) by these hydrogels followed pseudo-second-order kinetics and the Langmuir isotherm model, with maximum adsorption capacities of 114.94, 77.52, 138.89 and 75.19 mg/g for WT-PPH, AT-PPH, BTPPH, and PPCH, resspectively. These results indicated that separate bleaching treatment significantly improved the CR adsorption capacity.

Amsyar Ayob et al. (2020) further demonstrated the effectiveness of NaOH-treated pineapple waste-based adsorbents, achieving an 85.88 % removal efficiency for Pb^2+^ ions compared to 52.57 % for untreated adsorbents. The study also emphasized the importance of optimizing parameters, such as pH (4.0) and temperature (60 °C), to enhance the adsorption performance. Despite these promising results, the application of pineapple-waste-based biosorbents is still in its early stages, necessitating further research to elucidate the underlying mechanisms and optimize the processes for specific contaminants.

In recent years, aerogels have garnered attention as a class of three-dimensional (3D) materials characterized by high porosity and desirable properties for applications in air purification, water treatment, and catalysis. Pineapple peel-based oxidized cellulose nanofibrils (PP-TOCNF) were prepared through TEMPO-mediated oxidation and ball milling, yielding aerogels with lightweight properties, high porosity, excellent water absorption, and superior mechanical properties. These properties could be tailored by adjusting the oxidation degree of PP-TOCNF (Lv et al., 2022). Additionally, bacterial cellulose (BC), a biodegradable cellulose synthesized by bacteria such as *Gluconacetobacter xylinus*, has been explored for its high surface area-to-mass ratio, crystallinity, and physical and mechanical properties. BC has been widely used in the development of nanocomposites for applications in medicine, food, textiles, and environmental remediation (Katyal et al., 2023). Jittaut et al. (2023) reported a BC yield of 0.29 g/L using pineapple peel waste as a substrate, while Le et al. (2023) fabricated aerogels from bacterial cellulose derived from pineapple peel waste through freeze-drying. These aerogels exhibited a 3D network with large pores and a high density of surface hydroxyl groups, enabling effective adsorption of both cationic and anionic dyes.

In conclusion, pineapple by-products provide a sustainable and cost-effective solution for wastewater treatment, with significant potential for removing heavy metals, dyes, and other pollutants. However, further research is needed to optimize the modification processes, understand the adsorption mechanisms, and scale up the application of these materials for industrial use. The development of advanced materials such as aerogels and bacterial cellulose-based composites further expands the potential applications of pineapple by-products in environmental remediation.

## 5 Limitations of pineapple waste-derived pesticides

### 5.1 Variability in compound composition

Pineapple-waste-derived biopesticides offer a viable approach for implementing sustainable pest control methods. Nevertheless, the effectiveness of their performance is influenced by numerous factors, which require thorough evaluation and the use of standardized protocols to achieve optimal results. The formulation of biopesticides is a crucial factor that determines their efficiency. Varying formulations, such as extracts, oils, or emulsions, can impact the stability and diffusion of active chemicals, eventually influencing their efficacy in combating pests. The level of bioactive components in these formulations is crucial in determining their effectiveness.

The application method of biopesticides has a significant impact on their effectiveness. Variables such as the amount of spray used, the size of the droplets, and the frequency of application can impact how well the product covers and penetrates, ultimately affecting its effectiveness in controlling pests. Ensuring consistent application throughout targeted regions is essential for optimizing the efficacy of biopesticides and reducing the risk of pest control gaps.

The chemical makeup of biopesticides obtained from pineapple waste is naturally subject to variation (Daraban et al., 2023). Various factors, including the type of pineapple, extraction methods, and storage conditions, can all impact the composition and concentration of bioactive compounds. The diversity of biopesticide performance presents difficulties in maintaining consistency and predictability. Fenibo et al. (2022) emphasized the importance of this variability and its consequences for determining dosage and ensuring consistent application. The presence of variations in strength and efficacy among different batches highlights the importance of implementing strong standardization methods. Standardization guarantees that every batch of biopesticide maintains uniform potency and efficacy levels, therefore improving reliability and enabling more accurate pest control procedures.

### 5.2 Stability and shelf life

Phenolics, organic acids, and terpenes are bioactive compounds that play a crucial role in the formulation of many biopesticides. These compounds are highly effective in pest control due to their natural pesticidal properties. However, their efficacy is often compromised by their susceptibility to degradation under varying environmental conditions, such as fluctuations in light intensity, temperature, and pH levels (Qasim et al., 2024). This inherent instability significantly reduces the effective storage life and dispersion efficiency of compounded botanical pesticides, thereby limiting their long-term applicability in agricultural practices. Consequently, there is a pressing need to develop innovative strategies that enhance the stability and durability of these bioactives, ensuring their sustained effectiveness in pest management.

To address these challenges, significant advancements have been made in encapsulation technologies and formulation strategies. Encapsulation techniques, such as microencapsulation and nanoencapsulation, have emerged as promising solutions to protect bioactive molecules from environmental stressors and enzymatic degradation. These methods involve enclosing phenolics, organic acids, and terpenes within protective matrices, which act as barriers against adverse conditions. By doing so, encapsulation not only preserves the integrity of these compounds but also extends their shelf life and maintains their bioactivity over extended periods (Yeguerman et al., 2020). For instance, microencapsulation has been shown to enhance the stability of essential oils, which are rich in terpenes, by shielding them from oxidative degradation and evaporation (Pateiro et al., 2021). Similarly, nanoencapsulation has demonstrated the ability to improve the controlled release of phenolic compounds, ensuring their sustained efficacy in agricultural applications (Rezagholizade-shirvan et al., 2024).

In addition to encapsulation, novel delivery systems, such as slow-release formulations and biodegradable carriers, have been developed to further enhance the performance of botanical pesticides. Slow-release formulations enable the gradual release of active ingredients, providing prolonged pest control while reducing the frequency of application. This approach not only enhances the efficiency of the pesticide but also reduces the risk of environmental contamination (Boyandin et al., 2022). Biodegradable carriers, on the other hand, offer an eco-friendly alternative by breaking down into non-toxic byproducts after releasing the active ingredients. These carriers align with the principles of sustainable agriculture by reducing the ecological footprint of pesticide use (Bhaskar et al., 2024). The integration of these advanced technologies into the formulation of botanical pesticides offers multiple benefits. Firstly, it enhances the stability and longevity of bioactives, ensuring their effectiveness over time. Secondly, it improves the precision and efficiency of pesticide delivery, thereby reducing the quantity required for effective pest control. Lastly, these innovations contribute to the development of environmentally sustainable pest management practices, which are increasingly important in the context of global agricultural challenges (Isman, 2020).

In conclusion, the application of encapsulation techniques and advanced formulation strategies represents a significant step forward in the development of stable and effective botanical pesticides. By addressing the limitations posed by the inherent instability of phenolics, organic acids, and terpenes, these technologies pave the way for the widespread adoption of eco-friendly pest control solutions in agriculture. Future research should focus on optimizing these methods to further enhance their efficacy and scalability, ensuring their practical implementation in diverse agricultural systems.

### 5.3 Limited Spectrum of activity

Chemicals obtained from pineapple waste show potential effectiveness against specific pests and diseases. However, they tend to have a more limited range of action compared to conventional pesticides. Although these natural chemicals are effective against certain insects or pathogens, they are less effective against other types of pests, resulting in less than an ideal management of pests and diseases (Damalas & Koutroubas, 2020).

To improve their effectiveness and maximise overall efficiency, it is crucial to integrate combination formulations. These formulations could include synergistic chemicals that enhance the pesticidal activities of products made from pineapple waste. Furthermore, the incorporation of these natural substances with other methods of pest control can result in comprehensive and durable solutions. A comprehensive strategy for managing pests and diseases can be accomplished by combining chemicals obtained from pineapple waste with biological control agents such as fungal biopesticides, bacterial biopesticides, viral biopesticide and nanopesticide strategies. This comprehensive approach not only expands the range of effectiveness of pesticides obtained from pineapple waste but also decreases dependence on artificial chemicals, thereby promoting sustainable agriculture methods that are environmentally beneficial.

### 5.4 Cost-effectiveness and accessibility

While biopesticides have benefits for the environment and human health, their production costs often surpass those of conventional pesticides. The widespread use of these technologies is impeded by their restricted availability and cost-effectiveness, particularly in poor regions, notably in developing countries. The economic viability of pest management systems utilizing biopesticides depends on various factors, including the costs associated with manufacturing, processing, and application (Ngegba et al., 2022). To reduce manufacturing costs and enhance the accessibility of biopesticides derived from pineapple waste, it is essential to leverage economies of scale, government incentives, and public-private partnerships.

## 6 Regulatory considerations and future directions

The possibility of commercializing and expanding the use of pesticides obtained from pineapple waste is an interesting opportunity for the food industry. The economic viability of a pesticide made from pineapple waste depends on its effectiveness, accessibility, and positive environmental impact. Studies have shown that extracts derived from pineapple waste have strong pesticidal capabilities against various pests and diseases. Pineapple-growing areas generate a surplus of pineapple waste that can serve as an effective and sustainable resource for producing pesticides. These variables increase the possibility of developing manufacturing on a larger scale for commercial purposes.

By employing this waste, the burden on landfills is diminished, and the emission of greenhouse gases from waste decomposition is decreased. Moreover, the use of pineapple waste to produce pesticides provides farmers with an additional income stream, thereby promoting economic sustainability in pineapple-growing regions. This approach aligns with the principles of the circular economy and sustainable development by utilizing waste as a valuable resource. To effectively market pesticides derived from pineapple waste, it is imperative to tackle a range of issues. A significant impediment is the requirement for standardization and quality control protocols to ensure consistent efficacy and safety (Fouda-Mbanga & Tywabi-Ngeva, 2022). The heterogeneity in the composition of pineapple waste, the extraction processes employed, and the storage conditions can influence the efficacy of pesticides. To obtain regulatory approvals and achieve market acceptance, it is crucial to conduct comprehensive toxicity studies and comply with relevant regulatory frameworks (Wahab et al., 2022). To address these issues, it is essential to engage in interdisciplinary collaboration, conduct comprehensive research, and establish connections with the industry.

Recent research has contributed to the commercialization of pesticides derived from pineapple waste. Research has primarily focused on enhancing extraction methods to increase the yield and effectiveness of pesticides obtained. Additionally, researchers have assessed the efficacy of these pesticides against targeted pests using bioassays and field trials, providing valuable data for product development and registration. Advancements in formulation technologies, such as microencapsulation, enhance the stability and delivery of pesticides, hence prolonging their shelf life and effectiveness in the field. These research discoveries drive efforts to commercialize further.

Ultimately, the process of commercializing and expanding the production of pesticides produced from pineapple waste presents a hopeful alternative for achieving sustainable pest control in agriculture. Research findings, environmental advantages, and economic prospects align this method with the global agenda for sustainable development. Nevertheless, it is crucial to prioritize resolving issues related to quality control, regulatory compliance, and market acceptance. Ongoing study, innovation, and collaboration are essential to fully harness the potential of pesticides derived from pineapple waste in commercial agriculture.

## 7 Conclusion

Pineapple by-products are a rich source of bioactive compounds, including phenolics, terpenes, and organic acids, which exhibit pesticidal properties. These compounds can effectively repel or eliminate food storage pests, offering a sustainable solution for pest management. The repurposing of pineapple waste into valuable biopesticides aligns with the principles of the circular economy and waste valorisation. By transforming agricultural by-products into valuable resources, this approach reduces waste, enhances resource efficiency, and promotes sustainability in the food and agricultural sectors. This not only addresses pest management challenges but also contributes to reducing the environmental footprint of agricultural practices. The utilization of pineapple-derived bioactives offers a viable alternative to conventional pesticides. By integrating these eco-friendly solutions, agriculturists and food processors can reduce their dependence on chemical pesticides, thereby mitigating the risks associated with pesticide residues in food and the environment. This shift supports the global movement toward sustainable agriculture and safer food production systems.

## CRediT authorship contribution statement

**Isaac Duah Boateng**: Writing – review & editing, Validation, Supervision, Visualization. **Benjamin Bonsu Bruce:** Writing – review & editing, Writing – original draft, Visualization, Validation, Supervision, Conceptualization.

## Declaration of competing interest

The authors declare that they have no known competing financial interests or personal relationships that could have appeared to influence the work reported in this paper.

## Data Availability

No data was used for the research described in the article.
